# Free-Space Applications of Silicon Photonics: A Review

**DOI:** 10.3390/mi13070990

**Published:** 2022-06-24

**Authors:** Chung-Yu Hsu, Gow-Zin Yiu, You-Chia Chang

**Affiliations:** Department of Photonics, College of Electrical and Computer Engineering, National Yang Ming Chiao Tung University, Hsinchu 30010, Taiwan; cyhsu.eo07g@nctu.edu.tw (C.-Y.H.); gordon.eo07g@nctu.edu.tw (G.-Z.Y.)

**Keywords:** silicon photonics, LiDAR, optical phased array, beam steering

## Abstract

Silicon photonics has recently expanded its applications to delivering free-space emissions for detecting or manipulating external objects. The most notable example is the silicon optical phased array, which can steer a free-space beam to achieve a chip-scale solid-state LiDAR. Other examples include free-space optical communication, quantum photonics, imaging systems, and optogenetic probes. In contrast to the conventional optical system consisting of bulk optics, silicon photonics miniaturizes an optical system into a photonic chip with many functional waveguiding components. By leveraging the mature and monolithic CMOS process, silicon photonics enables high-volume production, scalability, reconfigurability, and parallelism. In this paper, we review the recent advances in beam steering technologies based on silicon photonics, including optical phased arrays, focal plane arrays, and dispersive grating diffraction. Various beam-shaping technologies for generating collimated, focused, Bessel, and vortex beams are also discussed. We conclude with an outlook of the promises and challenges for the free-space applications of silicon photonics.

## 1. Introduction

Silicon photonics is a reliable and scalable platform for integrating a large number of diverse waveguiding components on the same chip. It has reached successful commercialization in the transceiver market for data centers and 5G optical interconnects [1,2,3]. In most established data- and telecommunication applications, silicon photonic integrated circuits exploit guided modes rather than free-space radiation [4]. Recently, free-space applications of silicon photonics have emerged due to the increasing demands for the miniaturization of free-space optical systems down to the chip scale. Conventional free-space optical systems do not fulfill the requirements for autonomous cars, drones, and metaverse because of bulky sizes and the need for precise assembly [5]. These new applications urge innovative optical systems with reduced sizes, weight, and power consumption (SWaP). Notable examples include light detection and ranging (LiDAR) [6,7,8] and free-space optical communication (FSO) [9,10,11], where silicon photonics provides a promising alternative to replace the conventional bulky mechanical beam steerer with a chip-scale device that has no moving parts. A comprehensive optical system can be integrated on a tiny chip without the need for aligning and assembling the components. The number of components in a silicon photonic integrated circuit can easily scale up to hundreds or even thousands [12,13,14], allowing the creation of complex functionalities that are too difficult for conventional bulk optics. In particular, optical phased arrays (OPAs) [9,12,15,16], biophotonic sensors [17], and quantum photonic circuits [18] have leveraged the scalability.

A silicon photonic platform enables high-volume production by leveraging the mature monolithic fabrication process developed for decades in the semiconductor industry [19]. By using the complementary metal-oxide-semiconductor (CMOS) process, silicon photonics can share the fabless-foundry ecosystem. Many commercial foundries, including Taiwan Semiconductor Manufacturing Company (TSMC), GlobalFoundries, and Tower Semiconductor, support silicon photonic products. Designers can reliably develop their circuits using the process design kit (PDK) supplied by the foundries. Many high-performance components such as low-loss waveguides, phase shifters, high-speed modulators, grating couplers, and detectors are available in the PDK.

Silicon photonics is a competitive platform for miniaturing a free-space optical system. Compared with other integrated platforms such as InP, silicon photonics has a higher index contrast, thus allowing stronger confinement, tighter bends, and more compact chip areas. Silicon material allows p-type and n-type doping to create PN or PIN diode structures. By biasing the diodes, dynamic tuning of the silicon refractive index can be achieved by the plasma dispersion effect [20,21]. Silicon also has a large thermo-optic coefficient of 1.86 × 10^−4^ K^−1^ that allows index tuning [22,23]. Germanium can be grown epitaxially on selective silicon areas to serve as low bandgap materials for photodetection [24]. Electronic circuitry can be integrated monolithically with silicon photonics for analog and digital control [7,19]. Silicon photonics, therefore, has a comprehensive library to meet the various needs of building chip-scale free-space optical systems.

In this review, we will first introduce beam steering technologies enabled by silicon photonics in Section 2, including OPAs, focal plane arrays (FPAs), and dispersive grating diffraction. In Section 3, examples of integrated chip-scale optical systems realized with silicon photonics will be described, including LiDAR, FSO, and imaging systems. In addition, a substantial research effort is being dedicated to beam shaping with silicon photonics. Various free-space beams such as collimated beams, focused beams, Bessel beams, and vortex beams have been demonstrated, which will be discussed in Section 4. We will present other emerging free-space applications, including optogenetics and quantum photonics, in Section 5. Finally, in Section 6 we will provide an outlook on the promises and challenges.

## 2. Beam Steering Technologies

**Figure 1:**
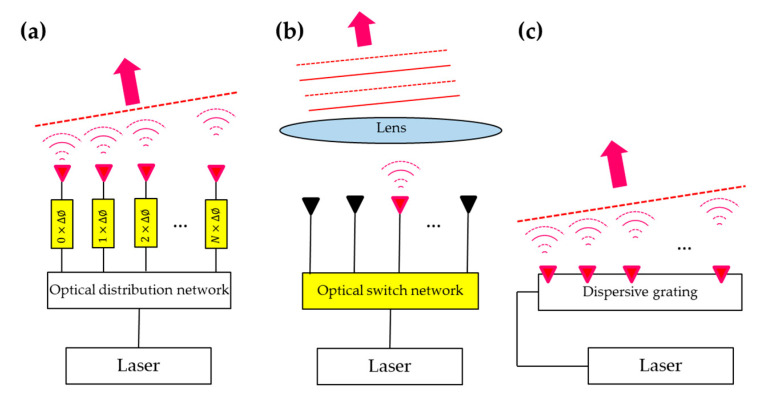
Three optical beam steering techniques: (**a**) optical phased array (OPA), (**b**) focal plane array (FPA), (**c**) dispersive grating diffraction.

Beam steerers play an essential role in many applications, including LiDAR, FSO [9], display [25], and laser-scanning microscopes [26]. A beam steerer scans a laser beam to different directions to detect objects or transfer optical power. Three major beam steering techniques have been explored in silicon photonics, including OPA, FPA, and dispersive grating diffraction, as depicted in Figure 1. A beam steerer can apply one technique on one axis and another on the second. In an OPA, as shown in Figure 1a, a coherent light source (typically a laser) is split to multiple waveguides with an optical distribution network. Light in each waveguide then goes through a phase shifter before reaching an emitter, where light exits the device. The emission from all emitters synthesizes a beam coherently while the direction is controlled by the overall phase profile. In an FPA, as shown in Figure 1b, the optical distribution network is replaced by an actively-controlled switching network. Light is switched to only one of the waveguide channels at a time. A lens collimates the emission and creates a beam in the far field. The collimated beam points in a different direction if the light is switched to a different emitter. In Figure 1c, beam steering is achieved by using a dispersive grating to diffract the light from the waveguide to the free space. The diffracted light has a deterministic wavelength-dependent direction. One can steer the diffracted beam by wavelength tuning of the laser source. We will discuss the principles and recent demonstrations of these three beam steering techniques in the following sub-sections.

### 2.1. Optical Phased Arrays

Phased arrays have been widely used in the microwave regime for beam steering, where the relative phase of an array of antennas is used to shape the radiation field through constructive and destructive interference [27]. In the optical regime, controlling the phases of a large number of emitters is challenging with bulk optics. Thanks to the capability of silicon photonics to integrate a large number of active components on the same chip, optical versions of phased arrays have been realized.

An OPA coherently synthesizes the emissions from individual emitters to form a low-divergence beam in the far field. By controlling the relative phases between individual emitters, the beam can be actively steered. The electric field of the emission from an OPA in the far field can be expressed as [28]:

(1) $$E\left(\theta ,\phi \right)=S\left(\theta ,\phi \right)\times AF\left(\theta ,\phi \right),$$

where $S\left(\theta ,\phi \right)$ is the far-field angular distribution of a single emitter. $AF\left(\theta ,\phi \right)$ is the array factor, which is given by:

(2) $$AF\left(\theta ,\phi \right)=\sum_{n=1}^{N}w_{n}\cdot e^{i2\pi x_{n}\cdot u}.$$

where $x_{n}$ is the position of the *n*th emitter, u=sinθcosϕ/λ, and $w_{n}=\left|w_{n}\right|e^{i\varphi_{n}}$ determines the phase and amplitude of the *n*th emitter. N is the number of emitters in the array. To synthesize a collimated beam pointing at the angle θ, the OPA is set to create a linear phase profile, as shown in Figure 1a. The phase difference between neighboring emitters is ∆*ϕ*. The array pitch is d. The pointing angle of the beam is given by:

(3) $$\theta =\sin^{-1}\left[\frac{\Delta \phi \;\lambda }{2\pi \;d}+m\frac{\lambda }{d}\right],$$

where m is an integer that represents the diffraction order. The field-of-view (FOV) is given by:

(4) $$\mathrm{FOV}=2\;\sin^{-1}\left(\frac{\lambda }{2d}\right).$$

Within the FOV, there is only one beam without ambiguous grating lobes. Notice that the FOV is determined by the array pitch d. If the amplitude $\left|w_{n}\right|$ of each emitter is equal, the far-field distribution of an OPA can be approximated by the diffraction of a slit of size Nd, which is given by:

(5) $$\mathrm{Far}-\mathrm{field}\;\mathrm{intensity}\;\mathrm{distribution}\;\propto \;\sin^{2}\left(\frac{\pi Nd}{\lambda }\theta \right)/{\left(\frac{\pi Nd}{\lambda }\theta \right)}^{2},$$

which has a full width at half maximum (FWHM) divergence angle of:

(6) $$\Delta \theta \approx \frac{2.78}{\pi }\frac{\lambda }{Nd}.$$

Notice that the divergence angle is determined by the total aperture size Nd of the emitter array. To produce a well-collimated beam that can propagate over a long distance in the free space, having a large emission aperture size is crucial. For beam steering applications, it is desirable to have a large FOV and a small beam divergence angle Δθ. The ratio between the FOV and Δθ is the number of resolvable angles for a beam steerer. To the first-order Taylor’s expansion, the number of resolvable angles is given by:

(7) $$\frac{\mathrm{FOV}}{\Delta \theta }\approx 1.13\;N.$$

Therefore, the number of resolvable points in the beam steering is approximately just the number of emitters N for an OPA with equally-spaced emitters. For high-resolution beam steering, it is crucial to building an OPA with a large number of elements. For example, large-scale OPAs with 512, 1024, and 8192 elements have been demonstrated in [12,13], respectively.

**Figure 2:**
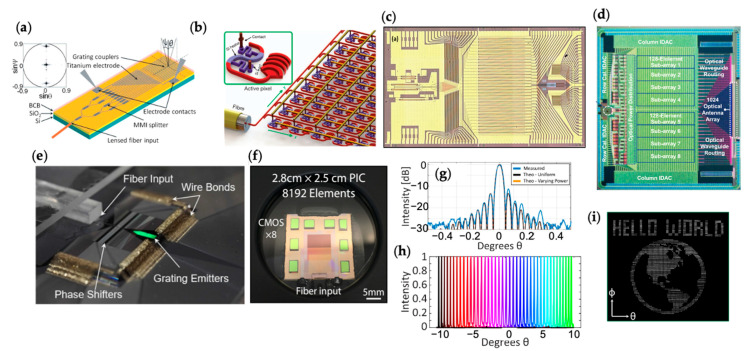
Implementations of silicon photonic OPAs. (**a**) Schematic of a 1D OPA combined with grating emitters. Reprinted with permission from [16] © The Optical Society. (**b**) Schematic of a 2D OPA. Reprinted with permission from Springer Nature: Nature [29] © Springer Nature (2013). (**c**) Optical image of a 32-element OPA on a heterogenous III-V/silicon photonic platform. Reprinted with permission from [15] © The Optical Society. (**d**) Optical image of a 1024-element OPA monolithically integrated with the electronics. © 2022 IEEE. Reprinted with permission, from [14]. (**e**) Optical image of a 512-element OPA wire-bonded to the driving electronics. Reprinted with permission from [12] © The Optical Society. (**f**) Optical image of an 8192-element OPA with co-packaged flip-chip CMOS drivers. Reprinted with permission from [13]. Copyright © 2022, Poulton et al. (**g**–**i**) Measured far-field angular distribution, beam steering to different angles, and scanning pattern from an OPA. Reprinted with permission from [9] © 2022 IEEE.

The implementations of silicon photonic OPAs for beam steering have been achieved with different variations. The common device structure of an OPA, as shown in Figure 1a, includes a passive optical distribution network, typically a tree of multimode interference (MMI) splitters or a star coupler, to distribute light to many waveguide channels. Each waveguide channel has an independent phase shifter to introduce a tunable phase shift to the channel. After passing the phase shifter, light emits to the free space by grating or edge couplers. An example of the silicon photonic implementations is shown in Figure 2a [16]. In this work, an MMI splitter tree is used to distribute the light to a 1D array of grating emitters. The Ψ direction is steered by the OPA mechanism, while the θ direction is steered by the dispersive grating diffraction. Figure 2b shows another example from [29], where a 2D optical distribution network is created with directional couplers. A 2D OPA is formed to steer in both directions with the active phase tuning. In this design, the array pitch is 9 µm × 9 µm in order to fit each unit cell with a phase shifter and a grating emitter. Therefore, the large array pitch limits the FOV, as described by Equation (4). Figure 2c shows the optical microscope image of a fabricated 32-element OPA on a heterogenous III-V/silicon photonic platform where lasers, amplifiers, photodiodes, phase shifters are integrated on the same chip [15]. Figure 2d shows a large-scale 1024-element OPA monolithically integrated with the electronics [14]. Figure 2e shows the photograph of a 512-element low-power multi-pass OPA wire-bonded with the driving electronics [12]. Figure 2f shows an 8192-element OPA with flip-chip-bonded CMOS digital-to-analog converter (DAC) electronics [13]. In Figure 2g,h, the angular distribution of a formed beam and the beam steering operation to different angles are demonstrated. These measurement results are from a 512-element OPA with an aperture area of ~10 mm^2^ in [9]. Figure 2i shows a scanning pattern obtained by this OPA. Most OPA demonstrations operate at the telecommunication wavelengths because of the transparency window of silicon and the mature components developed for communications. Recently, an active blue OPA operating at 488 nm has been demonstrated using the SiN platform [30].

**Figure 3:**
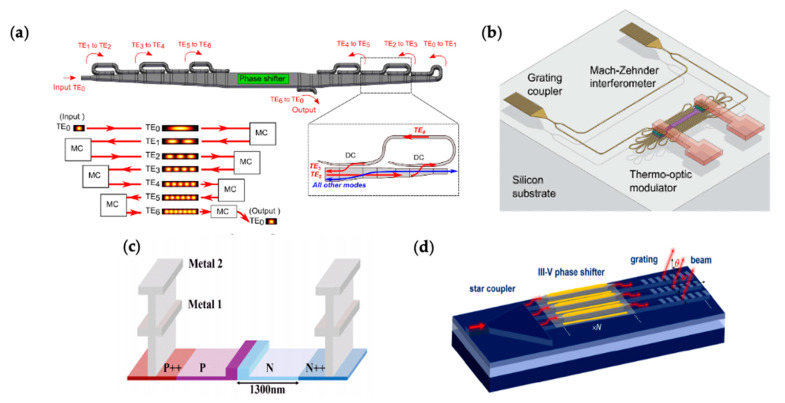
Various phase shifters employed in OPAs. (**a**) Multi-pass thermo-optic shifter [12]. (**b**) Thermo-optic phase shifter with dense dissimilar waveguide routing [36]. Reprinted with permission from [12,36] © The Optical Society. (**c**) Phase shifter based on the plasma dispersion effect. © 2022 IEEE. Reprinted with permission, from [38]. (**d**) Heterogeneous III-V/silicon phase shifter. Reprinted with permission from [39] © The Optical Society.

The phase shifter is the key enabling component in OPAs. Because an OPA may use hundreds or even thousands of active phase shifters, it is crucial to develop phase shifters with low power consumption, strong phase tuning, low crosstalk, and good reliability. Two major phase shifting mechanisms are primarily used—thermo-optic effect and electro-optic effect. Thermo-optic effect offers the strongest phase tuning among these mechanisms, thanks to the large thermo-optic coefficient in silicon (d*n*/dT = 1.86 × 10^−4^ K^−1^) [22]. It enables compact phase shifters that can be as short as several µm [29]. This mechanism is implemented in many OPAs [6,12,14,16,29,30,31,32,33,34,35]. However, a typical thermo-optic phase shifter has a $P_{\pi }$ of 10 s mW, where $P_{\pi }$ is the power required to create a phase shift of *π* [12]. For example, the 1024-element OPA in [14] consumes up to 55 W of power from the phase shifters. Several approaches have been developed to reduce the power consumption of the thermo-optic phase shifters. Miller et al. developed multi-pass mode-converting phase shifters with a $P_{\pi }$ of only 1.7 mW and implemented the phase shifters in a 512-element OPA [12], as shown in Figure 3a. Chung et al. used dense dissimilar waveguide routing to utilize the heat more efficiently, reducing the $P_{\pi }$ to 2.56 mW, as shown in Figure 3b [36]. On the other hand, the electro-optic effect mechanism enables phase shifters with low power consumption. The electro-optic effect in silicon can be attributed to the plasma dispersion effect [20,21] and the Kerr nonlinearity [37]. The electro-optic effect is weak in silicon, and, therefore, the phase shifters need to have sufficient lengths. A typical voltage-length product V·L is in the order of several V·cm [3]. There have been demonstrations of OPAs based on electro-optic phase shifters [9,13,38,39,40]. Figure 3c shows a schematic of a depletion-type electro-optic phase shifter used in an OPA that leveraged the plasma dispersion effect [38]. Poulton et al. demonstrated an electro-optic phase shifter with a maximum static power consumption of only 2 µW [9], which was orders of magnitude lower than thermo-optic phase shifters. Efficient electro-optic phase shifters can also be realized with heterogeneous integration of III-V materials on silicon. In addition to the plasma dispersion effect, III-V materials can include quantum-confined Stark effect (QCSE) or Franz–Keldysh effect, band filling effect, band shrinkage effect, and Pockels effect, to increase the overall electro-optic effect [39]. An implementation of OPAs on the heterogeneous III-V/silicon platform by Xie et al. is shown in Figure 3d [39]. The III-V/silicon phase shifter used in this work consumed a static power of less than 3 nW.

**Figure 4:**
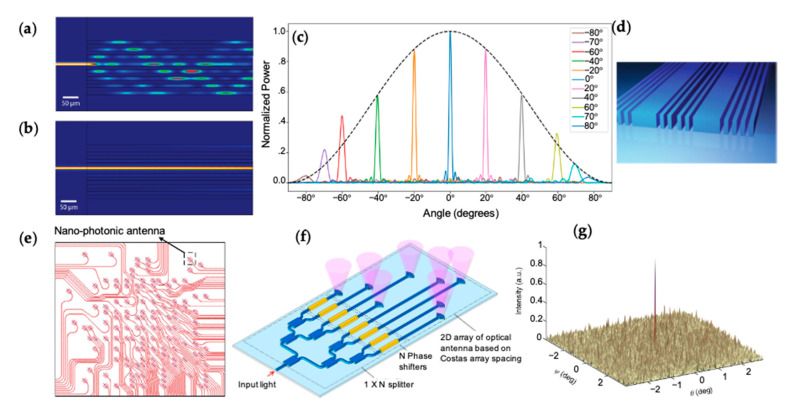
(**a**) Simulation of light propagation in an array of waveguides with equal width of 400 nm on λ/2 pitch [41]. (**b**) Simulation of the phase-mismatched waveguide array with sequentially varying widths of 300, 350, and 400 nm [41]. (**c**) Measured far-field optical power versus output angle over a 180° FOV. Reprinted with permission from [41]. Copyright © 2022, Phare et al. (**d**) Control of the evanescent field by all-dielectric metamaterial between waveguides [42]. Copyright © 2022, Jahani et al. (**e**) A non-uniform 2D sparse OPA with 128 elements. © 2022 IEEE. Reprinted with permission, from [35]. (**f**,**g**) Schematic of a 2D Costas array and the measured far-field beam profile. Adapted with permission from [32] © The Optical Society.

To further improve beam steering performances, such as the FOV and the number of resolvable points, different novel variations of OPAs have been investigated. To increase the FOV, an OPA needs to pack the waveguides closely to reduce the array pitch, as given by Equation (4). However, light in one waveguide can couple to nearby waveguides if a small array pitch is used, as shown by the simulation in Figure 4a [41]. Phare et al. addressed the coupling issue by using phase-mismatched waveguides with varied waveguide widths (Figure 4b). They demonstrated an OPA with a λ/2 pitch and a FOV of 180 degrees (Figure 4c) [41]. Other techniques to reduce the coupling between closely-packed waveguides include using metamaterials to engineer the evanescent tail of the waveguide mode (Figure 4d) and optimizing the waveguide thickness [31,42]. The techniques discussed above increase the FOV, but the number of resolvable points remains approximately N, as precited by Equation (7). One can circumvent this limitation by using non-uniform arrays. Non-uniform array pitches can suppress the grating lobes to obtain the full FOV at the cost of an increase in background noise [32,33,35]. It is possible to obtain a greater number of resolvable points than N. For example, in [35], a non-uniform 2D sparse OPA with 128 elements achieved at least 400 resolvable points (see Figure 4e). Fukui et al. used a 2D Costas array to obtain ~19,000 resolvable points with 127 elements, as shown in Figure 4f,g [32]. One can see the formation of a high-resolution beam and the increased background noise in Figure 4g.

### 2.2. Focal Plane Arrays

**Figure 5:**
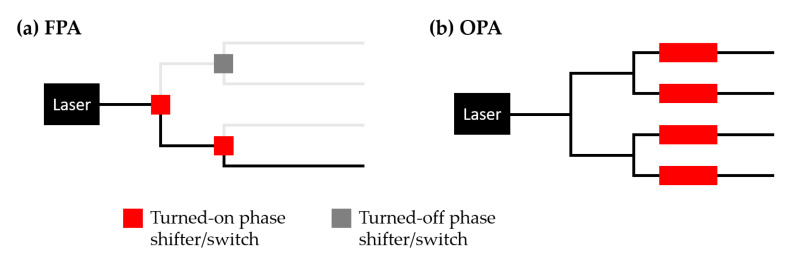
Comparison of the electric power consumption between the focal plane array (**a**), and the optical phased array (**b**).

An FPA-based beam steerer consists of a switchable emitter array and a lens [7,43,44,45,46,47], as shown in Figure 1b. The operation principle was explained at the beginning of Section 2. The number of switchable emitters directly determines the number of resolvable points. To reduce the beam divergence angle, one needs to keep a small emission area and reduce the aberration of the collimation lens [43,44]. Compared with OPAs, an FPA beam steerer requires much less electric driving power [44]. This is because only the switches along a single path need to be turned on, as shown in Figure 5. In contrast, light in an OPA is distributed to all paths, and all phase shifters need to be turned on all the time to maintain a deterministic emitting phase profile. The power consumption of an FPA beam steerer is proportional to $Log_{2}N$, which is a more favorable scaling compared with the N scaling of an OPA beam steerer [44]. FPA beam steerers do not require the complicated phase calibration procedures used in OPAs [48,49,50]. However, there are blind angles because the steerable angles of an FPA beam steerer are discrete [46].

**Figure 6:**
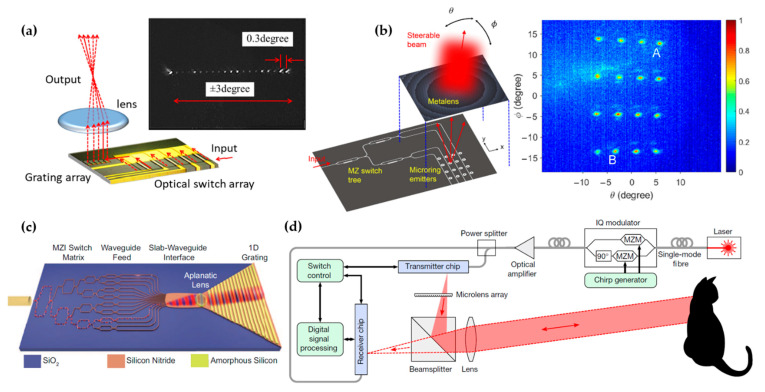
Demonstrations of active beam steering based on the FPA approach. (**a**) Schematic and beam steering result of a 1D FPA with a singlet lens. Adapted with permission from [43] © The Optical Society. (**b**) Schematic and beam steering result of a 2D FPA with a metalens and a microring emitter array. Reprinted with permission from [44] © The Optical Society. (**c**) FPA with an integrated planar lens. Reprinted with permission from [45]. Copyright © 2022, López et al. (**d**) LiDAR system that implements FPA for beam steering. Reprinted with permission from Springer Nature: Nature [7], © Springer Nature (2021).

Figure 6a shows a 1D FPA beam steerer demonstrated in [43]. The array consists of 20 grating emitters arranged in one dimension. A singlet lens is placed on top of the chip to collimate the light emission from the emitters array. A dense array with a smaller emitter area is desired to achieve high resolution. In this work, the authors put effort into reducing the grating emitter dimension to 14 µm. A FOV of ±3° was demonstrated. However, beam distortion due to the aberration of the lens was observed at large angles.

To allow a larger FOV and a more compact size, Chang et al. demonstrated an FPA beam steerer that used a metalens for collimation [44], as shown in Figure 6b. The metalens consisted of subwavelength-spaced nanopillars, allowing arbitrary tailoring of the lens phase profile. The authors engineered the phase profile to be aberration-free up to ±13.6°. The metalens had a flat footprint and a height of only 990 nm, which was more compact than conventional bulky lenses. The authors achieved 2D beam steering with a tightly-packed 2D switch network based on microring emitters. They demonstrated steering of 4 × 4 directions with a FOV of 12.4° × 26.8° and an FWHM divergence angle of 0.8°.

In [45], an FPA beam steerer with a planar lens integrated on the silicon photonic chip was demonstrated (Figure 6c). The planar lens confined the light in a slab waveguide and collimated the propagation direction of the guided wave. The authors showed a 1D FPA with 16 channels and a full FOV of 38.8°. The 1D FPA was combined with the dispersive grating diffraction approach to achieve beam steering in both directions. This fully-integrated design enabled a flat footprint without the need for assembling an external lens. However, the planar lens showed a severe aberration. In the following numerical work, the authors proposed a photonic crystal Luneburg lens to correct the aberration by up to ±80° [47].

FPA-based beam steering was recently implemented to create a frequency-modulated continuous-wave (FMCW) LiDAR system [7]. As shown in Figure 6d, both the transmitter and receiver chips used FPA for beam steering. The on-chip switch network of the FPA was combined with a local oscillator (LO) to achieve coherent heterodyne detection of the distance and velocity. This LiDAR system allowed scanning of 32 × 16 pixels.

### 2.3. Dispersive Grating Diffraction

Diffraction from grating couplers can be leveraged to perform beam steering with wavelength tuning. Grating couplers are periodic structures that exhibit angular dispersion (Figure 1c). The diffraction angle of the out-coupled light from the waveguide to the free space is governed by the Bragg condition [51]:

(8) $$\sin\theta =n_{eff}-\frac{\lambda }{\Lambda },$$

where *θ* is the diffraction angle from the grating, $n_{eff}$ is the effective refractive index of the guided mode, and Λ is the grating period. From Equation (8), the diffraction angle *θ* can be steered by varying the laser wavelength. Notice that the $n_{eff}$ is also wavelength-dependent. To achieve a large FOV, one needs a strong wavelength sensitivity, which is given by:

(9) $$\frac{d\theta }{d\lambda }=\frac{-1}{\cos\theta }\left(\frac{n_{g}-n_{eff}}{\lambda }+\frac{1}{\Lambda }\right),$$

where $n_{g}$ is the group index.

**Figure 7:**
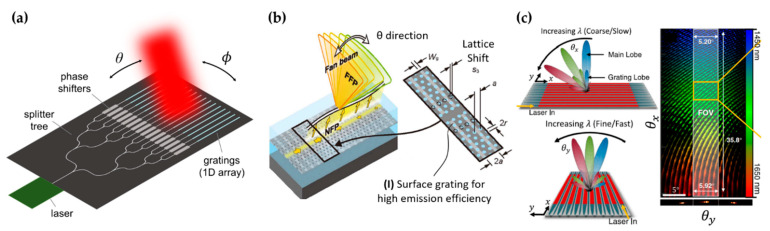
(**a**) Schematic of a 2D beam steerer that uses OPA and dispersive grating diffraction to steer in the ϕ and θ directions, respectively. Adapted with permission from [12] © The Optical Society. (**b**) Schematic of a beam steerer based on the enhanced angular dispersion of gratings on photonic crystal slow-light waveguides. Adapted with permission from [52] © The Optical Society. (**c**) A beam steerer that steers in both dimensions with dispersive grating diffraction. Adapted with permission from [55] © The Optical Society.

Beam steering based on dispersive grating diffraction can combine with another independent mechanism such as OPA or FPA to achieve 2D steering [12,16,52]. For example, in the device shown in Figure 7a, the beam is steered by the OPA in the ϕ direction and by the dispersive grating diffraction in the θ direction [12,53]. The typical wavelength sensitivity of grating diffraction, as in [12], is 0.265°/nm. The FOV is limited by the wavelength sensitivity and the tuning range of the laser. In [12], the authors obtained an FOV of 6° using a wavelength tuning Δλ of 23 nm. The minimum divergence angle is determined by the length of the grating emission. A weak grating with a long emission length can produce a narrow divergence angle in the far field [54]. We will elaborate on this in the beam shaping discussion in Section 4.1.

Dispersive grating diffraction usually has a small FOV due to the low wavelength sensitivity. To enhance the wavelength sensitivity, Ito et al. employed the slow-light effect in photonic crystal waveguides [8,52,56,57,58]. This strategy can be understood by Equation (9), where a large $n_{g}$ in slow-light waveguides can increase the sensitivity. In [52], a lattice-shifted photonic crystal waveguide (LSPCW) with shallow etched grating was used to create steerable light emission (Figure 7b). The authors achieved a sensitivity of ≈1°/nm. Using a wavelength tuning Δλ of 23 nm and a customized prism lens to exploit both the positive and negative diffraction angles, the authors demonstrated a large FOV of ±20°. They also showed 2D beam steering by combining the dispersive grating diffraction with the FPA mechanism, where a full FOV of 4.4° was achieved in the second axis.

Gratings can be arranged to exhibit angular dispersion in two dimensions to achieve a passive 2D beam steerer driven by wavelength sweeps, as demonstrated by Dostart et al. [55] (Figure 7c). The gratings were connected into a serpentine structure such that the diffraction angles $\theta_{x}$ and $\theta_{y}$ were both wavelength-dependent. The beam steerer achieved an FOV of 36° × 5.5° using a wavelength sweep of 1450–1650 nm. The serpentine grating structures could be tiled together to form a large-area emitting aperture with a high filling factor of 0.82. This is particularly important for low-divergence and long-distance steering applications because the aperture area determines the divergence angle.

Due to its simplicity and passive nature, the dispersive grating diffraction mechanism can be flexibly combined with wavelength multiplexing techniques to perform parallelism [58,59]. For example, in [59], Riemensberger et al. used a Kerr frequency comb light source from a SiN microring to obtain high throughput ranging and sensing.

## 3. Integrated LiDAR, Single-Pixel Imaging, and FSO System

Silicon photonics enables the integration of various functional components on a monolithic platform, providing a path to miniaturizing an optical system to a chip scale [19,60]. Monolithic integration has proved to be a powerful approach to the design and manufacture of optical transceivers [1,2,3]. An optical communication system composed of modulators, multiplexers/demultiplexers, photodetectors, etc. can be manufactured on the same chip without the need for alignment and package. Recently, there have been demonstrations of monolithically-integrated optical systems for free-space applications, including LiDAR [6,7,8,61], imaging systems [62,63], and FSO systems [9,10,11,64]. These integrated free-space optical systems offer competitive sizes, weights, and cost, over conventional bulk optics.

**Figure 8:**
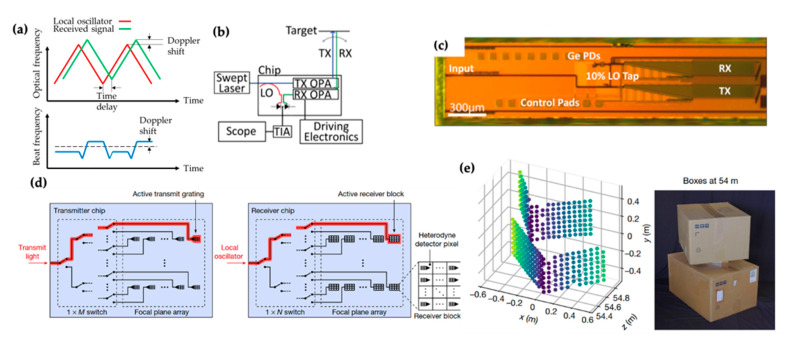
(**a**) Operation principle of the FMCW ranging technique. (**b**,**c**) Schematic and device optical image of an OPA-based FMCW LiDAR system. Reprinted with permission from [6] © The Optical Society. (**d**,**e**) Schematic and measured point cloud of an FPA-based FMCW LiDAR system. Another schematic of this system is shown in Figure 6d. Reprinted with permission from Springer Nature: Nature [7], © Springer Nature (2021).

A silicon photonic LiDAR system consists of two main functions: beam steering and ranging [65,66,67]. We discussed various beam steering technologies available in silicon photonics in Section 2. Ranging in a silicon photonic LiDAR is often achieved with the FMCW technique [5,68]. In contrast to time-of-flight (TOF) ranging [5], where the distance is calculated from the return time of reflected optical pulses, FMCW uses continuous waves. Continuous waves are preferred in silicon photonics because the strong peak power of optical pulses can cause two-photon absorption and the subsequent free-carrier absorption in silicon waveguides [24]. The mechanism of FMCW is depicted in Figure 8a [5,68]. By using a frequency-chirped laser source, the light reflected from an object is mixed with the LO to create a beat signal. The frequency of the beat signal contains the information of the distance and the velocity of the object. Compared with the incoherent TOF technique, FMCW technique uses coherent detection mechanism, which has the advantages of achieving photon shot noise-limited detection and immunity to photon backgrounds. The velocity can be obtained directly from the Doppler shift. FMCW can also use lower-bandwidth electronics than the TOF technique.

An example of an integrated LiDAR system is shown in Figure 8b,c [6]. It uses a transmitting OPA and a receiving OPA for beam steering. A 10% portion of the input swept laser source is tapped to serve as the LO and mixes with the received power to allow FMCW ranging detection. In this work, the optical components for beam steering, FMCW ranging, and photodetection are all integrated monolithically on a silicon photonic chip with a size of 6 mm × 0.5 mm. The authors achieved distance and velocity measurement with a range of 2 m and a resolution of 20 mm. Figure 8d,e show another integrated LiDAR system based on FPA beam steering and FMCW ranging [7]. The corresponding system diagram was introduced in Figure 6d. In this work, the silicon photonic chip was fabricated in a commercial foundry and leveraged the monolithic integration of the photonic and electronic circuits. The electronic circuits, including the balanced detectors, transimpedance amplifiers, and filters, were monolithically integrated with the receiver pixels to overcome the previous difficulties in providing electrical and photonic connections to each pixel. This LiDAR system had 32 × 16 pixels and achieved an accuracy of 3.1 mm at a distance of 75 m using 4 mW of optical power. The measured point cloud of an object at 54 m is shown in Figure 8e.

**Figure 9:**
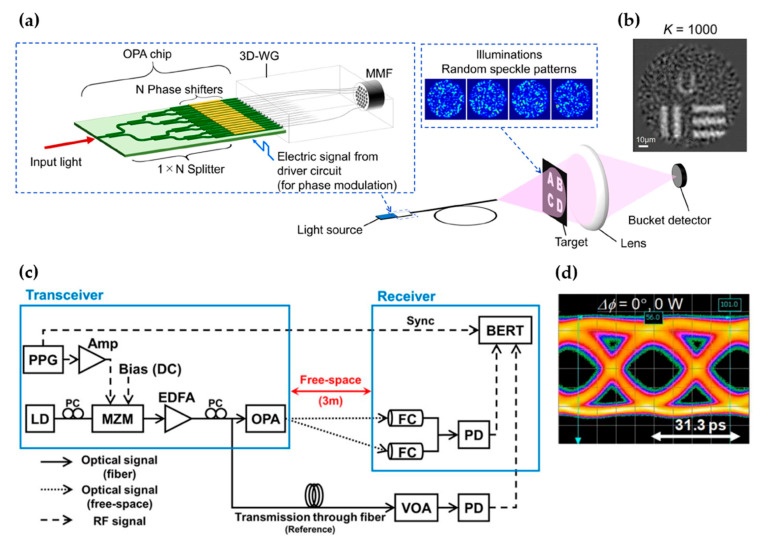
(**a**,**b**) Schematic and measured image of a single-pixel imaging system. Reprinted, with permission, from [62] © 2022 IEEE. (**c**,**d**) Schematic and measured eye diagram of an FSO system. Reprinted, with permission, from [11] © 2022 IEEE.

In addition to LiDARs, silicon photonics enables other free-space optical systems for single-pixel imaging [62,63] and FSO [9,10,11]. Figure 9a,b show an integrated single-pixel imaging system demonstrated by Fukui et al. [62]. The authors guided the output light from an OPA to a multimode fiber by 3D-printed waveguides. By controlling the OPA, a series of random speckle patterns were generated at the output of the multimode fiber and were used to illuminate a target. These patterns allowed imaging the target with a single-pixel detector, as shown in Figure 9b. This system achieved 1007 resolvable points with only 128 phase shifters. Figure 9c shows the schematic of an FSO system using an OPA together with thermo-optic tunable gratings for steering from the transmitter to the receiver [11]. This work demonstrated error-free transmission of 32 Gbps data over a distance of 3 m. The beam steering FOV was 46.0° (10.2°), and the divergence angle was 0.7° (0.9°) in the transversal (longitudinal) direction.

## 4. Beam Shaping

### 4.1. Beam Expansion and Focusing

Many free-space applications such as LiDAR and FSO require sending a collimated beam to a distant object. To achieve efficient delivery of optical power, reducing the divergence angle is essential. The mode size of the collimated beam determines the divergence angle. This can be seen in our discussion of OPAs, where Equation (6) states that a low-divergence OPA emission requires a large aperture size. In fact, the inverse proportionality between the divergence angle and the mode size is a fundamental diffractive nature of light given by the uncertain principle of waves. For example, a Gaussian beam has a $1/e^{2}$ divergence angle of $\lambda /\left(\pi w_{0}\right)$, where $w_{0}$ is the beam size at the waist [69]. To create a low-divergence beam that can propagate over meters of distance, the mode size needs to be at least in the order of millimeters. For a Gaussian beam with a waist diameter $2w_{0}$ of 1 mm and a wavelength of 1550 nm, the Rayleigh range $\left(\pi w_{0}^{2}\right)/\lambda$ equals ~0.5 m. However, creating a large-area emission from a silicon photonic chip is challenging. The typical single-mode silicon strip waveguide is only 450 nm × 220 nm, which is more than three orders of magnitude smaller than a millimeter beam. Therefore, there have been considerable efforts in the community to achieve beam expansion for a long-length or a large-area emission [39,54,70,71,72].

**Figure 10:**
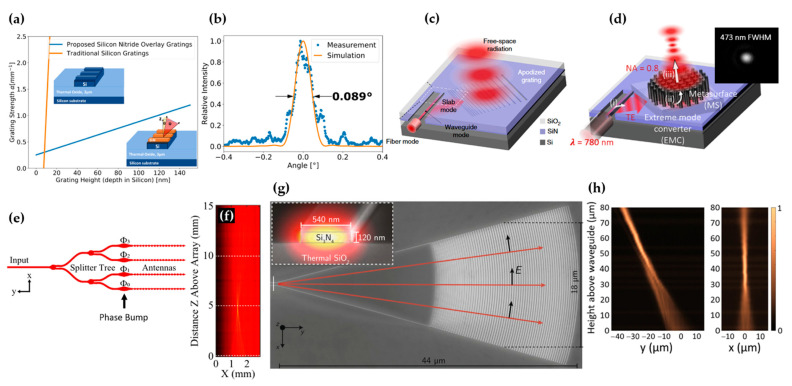
(**a**) Simulation of the grating strength of a grating formed by etching into a 250 nm × 450 nm silicon waveguide (orange) and by etching a 120 nm SiN overlay on the same silicon waveguide (blue) [54]. (**b**) Far-field measurement of a 1 mm-long SiN-overlay grating with an apodized duty cycle. Reprinted with permission from [54] © The Optical Society. (**c**) Schematic of a 2D beam expander [70] © 2022, Kim et al. (**d**) Beam focusing using a metalens on top of a 2D beam expander. Inset: optical image of the focused spot. Adapted with permission from [73] © 2022 American Chemical Society. (**e**,**f**) Schematic and measured cross-sectional intensity of an OPA designed for beam focusing. Reprinted, with permission, from [76] © 2022 IEEE. (**g**,**h**) SEM image and measured beam profiles of a grating coupler designed for beam focusing [79] © 2022 Mehta et al.

In the work by Zadka et al. [54], a waveguide grating with a SiN overlay was used for light diffraction to achieve a millimeter-long emission. The key is to introduce a perturbative structure that enables a weak grating strength on the order of mm^−1^ in a controllable manner. As shown in Figure 10a, the SiN overlay grating had a much weaker grating strength than the conventional silicon grating due to the lower refractive index contrast. The SiN overlay grating also had a lower sensitivity to the grating height, which enabled better fabrication tolerance. The authors apodized the grating strength by controlling the duty cycle. They demonstrated a super-Gaussian emission profile in 1D and measured a far-field divergence angle of 0.089° (Figure 10b). Kim et al. demonstrated beam expansion in 2D, creating a Gaussian beam with a waist $w_{0}$ of ~160 µm [70]. This corresponded to an increase in the modal area by a factor of >10^5^. The mode expansion was achieved in two separate stages, as shown in Figure 10c. The mode in a strip waveguide was expanded in 1D by evanescent coupling to a slab waveguide. An apodized grating was used to expand the mode in the second dimension.

Shaping the free-space emission from a silicon photonic chip to create a focused beam has been achieved with different approaches [18,73,74,75,76,77]. Beam focusing allows optical excitation or sensing exclusively of a small volume in the space, which is critical for quantum computation with ion qubits [18], optical tweezers [78], optogenetics [17], FPA-based beam steerers [44], and microscopy. To create a focused beam, one needs to engineer the phase profile of the emission. In the work by Yulaev et al. [73], the authors added a metalens on top of the 2D beam expander demonstrated in [70], as shown in Figure 10d. Working on the SiN platform, they generated a focused spot with a diffraction-limited size of 473 nm at the wavelength of 780 nm. Such a metasurface approach was pushed a step forward by integrating metasurfaces monolithically on silicon waveguides [75]. An OPA can also be designed to emit a focused beam, as demonstrated by Notaros et al. (see Figure 10e,f) [76]. The authors used the size of phase bumps to introduce a pre-determined phase delay in each waveguide channel. A focusing phase front was passively achieved. They demonstrated a focused spot with an FWHM size of ≈6.4 µm at the wavelength of 1550 nm. In the work by Mehta et al. [18,79], grating couplers were designed to create a focused emission (see Figure 10g,h) to deliver light to individual trapped ions for quantum manipulations and readouts. They apodized the grating strength and emission angle by changing the duty cycle and the period, respectively, creating a Gaussian amplitude profile and a focusing phase profile. The fabricated SiN grating couplers produced diffraction-limited spots with 1/*e*^2^ radii of 2.0 μm (x direction) and 2.3 μm (y direction) at λ = 674 nm. A systematic approach to design apodized gratings for focusing and other general profiles was developed recently by Zhao et al. [80].

### 4.2. Bessel Beams and Orbital Angular Momentom Beams

**Figure 11:**
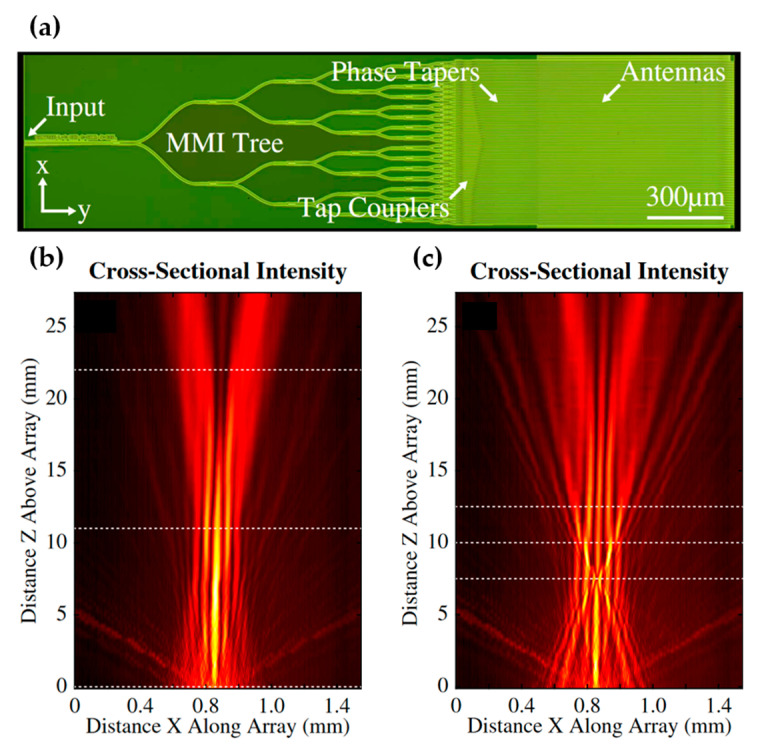
(**a**) Optical image of an OPA for quasi-Bessel beam generation [82]. (**b**) The measured quasi-Bessel beam from the OPA in (**a**). (**c**) The measured intensity distribution of a quasi-Bessel beam blocked by a gold wire. Reprinted with permission from [82] © The Optical Society.

Bessel beam has attracted attention due to its non-diffractive and self-healing properties and has found important applications in microscopy [81]. Free-space emission from a silicon photonic chip can be shaped to emit Bessel beams. In the work by Notaros et al. [82], a quasi-Bessel-beam was generated using a passive OPA. The phase and amplitude of the emission were controlled by the phase tapers and tap couplers, as shown in Figure 11a. The authors demonstrated the formation of a quasi-Bessel-beam (Figure 11b) and its self-healing ability by blocking the beam with a gold wire (Figure 11c).

**Figure 12:**
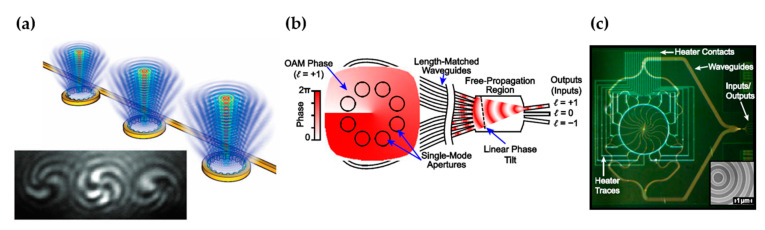
(**a**) Schematic of three OAM beam generators based on mirroring resonators with angular gratings (top). The bottom figure shows the interference pattern between three OAM beams (*l* =−3) and a co-propagating right-handed circularly-polarized beam. From [84]. Reprinted with permission from AAAS. (**b**,**c**) Schematic and optical images of an OAM beam encoder/decoder based on a silicon photonic integrated circuit. Reprinted with permission from [10] © The Optical Society.

A beam that carries orbital angular momentum (OAM) can provide another degree of freedom for transmitting more information [83]. Silicon photonic platform can enable compact devices to emit free-space beams with different OAM states [10,84,85,86,87,88]. In the work by Cai et al. [84], microring resonators with angular gratings were used to generate vortex beams, as shown in Figure 12a. The emission from the device was predominantly azimuthally-polarized and carried different OAM states, forming a vector vortex centered at the microring. This vortex beam emitter had a compact size with a radius of 3.9 µm. The microring-based device designs were employed for multiplexing in FSO [85] and quantum information [86]. Another design that uses a silicon photonic integrated circuit to produce OAM states was developed by Su et al. [10]. The schematic and image of the fabricated device are shown in Figure 12b,c. The design principle was similar to an OPA, where light was split to multiple waveguide channels. Each channel was length-matched, and the phase was corrected by heaters. The authors demonstrated free-space spatial-division-multiplexing (SDM) optical transmission with OAM states over a topological charge range of −2 to +2.

## 5. Other Emerging Free-Space Applications

Silicon photonics has been applied to optogenetics and trapped-ion quantum photonics to provide precise free-space light delivery. In these applications, light emits from the chip to the free space at precise positions to access the neurons or qubits. The mature CMOS fabrication allows the construction of large-scale reconfigurable circuits to address a large number of neurons or qubits, which is challenging with conventional bulk optics. The wavelengths are chosen carefully to stimulate the modified neurons or to drive the optical transition of qubits [17,18]. For example, 473 nm was used in [17] for neuron stimulation, and 674 nm was chosen in [18] for accessing ^88^Sr^+^ trapped ion qubits. Because silicon is not transparent below the wavelength of 1100 nm, another CMOS-compatible material, SiN, is employed [30,89,90,91].

**Figure 13:**
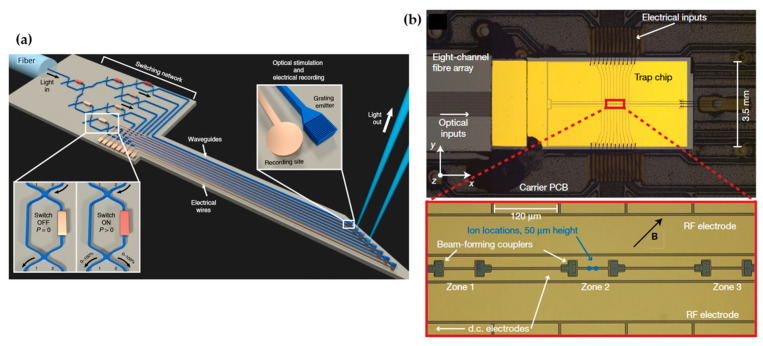
(**a**) Schematic of an optogenetics probe based on the SiN platform. Reprinted by permission from Springer Nature: Nature Biomedical Engineering [17] © Springer Nature (2020). (**b**) Optical micrograph of a photonic circuit integrated with a surface-electrode ion trap to obtain quantum logic gates. Reprinted by permission from Springer Nature: Nature [95] © Springer Nature (2020).

In optogenetics, blue light is typically used to stimulate neurons that are modified to express light-sensitive proteins. To study the brain circuitry, it is important to deliver light precisely to a small population of neurons deep in the brain. Silicon photonics can provide an implantable, precise, switchable light delivery system [17,92,93,94]. Mohanty et al. demonstrated an implantable reconfigurable probe based on the SiN platform [17], as shown in Figure 13a. The probe allowed stimulation of neurons across deep brain regions with a switching speed of <20 µs. Laser at 473 nm wavelength was precisely delivered to specific positions through a switching network. Light emitted to the free space from the gratings and stimulated specific neutrons. The optical stimulation was combined with nearby tungsten electrodes to record the neuron activity. Implantable silicon photonic light delivery probes have also been used for fluorescence imaging [93,94]. For example, Sacher et al. developed implantable probes to generate light-sheets deep in scattering tissues and demonstrated in vitro and in vivo fluorescence imaging of mouse brains [93].

For quantum information processing, the SiN platform can offer a scalable and robust light delivery system for trapped ion qubits. As shown in Figure 13b, a photonic circuit integrated with a surface-electrode ion trap was demonstrated by Mehta et al. [95]. Light at 729 nm wavelength was routed by the waveguides to precise positions, where grating couplers created focused emission through the free space to address the trapped ions. This waveguide approach eliminated beam pointing instabilities encountered by bulky optics and reduced drifts and noises. It also allowed parallelism for future scaling to a system with a larger number of qubits.

## 6. Outlook

Silicon photonics has shown great promise in integrating an optical system on a chip for free-space applications. Some examples have shown early successes, such as beam steering devices for LiDAR and light delivery circuits for quantum photonics. However, some key performances of silicon photonic systems, such as optical losses, are still far behind conventional bulk optics. Several challenges remain to be addressed.

First, optical losses in waveguides and waveguide-based components are much higher than free-space bulk components. Free-space bulk components such as lenses are usually made of glasses or plastics. Light propagates in air and these materials with negligible optical losses. With anti-reflective coatings, free-space bulk components can achieve <0.5% loss per surface from Fresnel reflection. Dielectric mirrors have high reflectivity of >99%. These anti-reflective and high-reflective coatings can be further optimized for higher demands. In contrast, silicon waveguides are lossy. The typical propagation loss of strip silicon waveguides reported by foundries is 1–2 dB/cm [60], which limits the maximum length one can design for a waveguide-based optical system. The propagation loss is mostly due to the scattering from the waveguide sidewall roughness, which depends on the fabrication. One potential solution is to work with lower-index-contrast SiN waveguides [96], which are also available in the CMOS process. Another major source of optical loss occurs at the coupling between a fiber and a silicon photonic chip. Fiber coupling is implemented mostly with either grating couplers or inverse tapers. Grating couplers typically cause <3 dB/facet [60,97]. Inverse tapers have slightly better coupling efficiencies but still have a typical loss of ~1 dB/facet [19,98]. Improving the waveguide propagation losses, fiber-waveguide coupling losses, and the efficiency of various waveguide-based components are crucial for silicon photonics to compete with conventional free-space bulk optical systems.

One of the most attractive features of applying silicon photonics to free space is the ability for active tuning. It enables an optical system with sophisticated, active functionalities such as beam steering. However, the use of a large number of active components, such as phase shifters, would consume huge electrical driving power [12]. As discussed in Section 2.1, novel efficient phase shifters are currently under intensive research [12,39]. Wiring and routing of the electrical wires represent another challenge when integrating a large number of active components, which can potentially be mitigated by monolithic integration of CMOS driving electronics with photonics [7,19].

For applications such as LiDARs that require sending a free-space beam to a distant object, it is crucial to have a large beam size, as discussed in Section 4.1. However, there is a huge mode–size mismatch between a silicon photonic waveguide and a well-collimated free-space beam. A silicon photonic waveguide confines the light to a subwavelength mode size by the high refractive index contrast. To create a reasonably collimated beam that can propagate over at least meters for LiDAR applications, the mode size needs to be at least millimeters, which is more than three orders of magnitude larger than the waveguide mode. It is challenging to expand a mode by such a huge factor. OPAs address this challenge by using many waveguide emitters to synthesize a large aperture [13]. Other approaches include making weak gratings [39,54,71] or designing evanescently-coupled mode-size converters [70]. Further research on reliable mode expansion is of key importance.

Silicon photonics is poised to change the landscape of optical systems. It has shown impressive success in fiber telecommunications [1,2,3], and we expect to see more and more applications in the free space. Silicon photonics enables miniaturization to the chip scale, large-scale integration of many components, reconfigurability with active tuning, and mass production with high yield. These unique advantages of silicon photonics will open up new possibilities for next-generation free-space optical systems.

## Data Availability

Not applicable.

## References

[1] Subbaraman H., Xu X., Hosseini A., Zhang X., Zhang Y., Kwong D., Chen R.T. (2015). Recent Advances in Silicon-Based Passive and Active Optical Interconnects. Opt. Express.

[2] Cheng Q., Bahadori M., Glick M., Rumley S., Bergman K. (2018). Recent Advances in Optical Technologies for Data Centers: A Review. Optica.

[3] Chang Y.C., Lipson M. (2020). Nanophotonic Devices for Power-Efficient Communications. Optical Fiber Telecommunications VII.

[4] Mekis A., Gloeckner S., Masini G., Narasimha A., Pinguet T., Sahni S., De Dobbelaere P. (2011). A Grating-Coupler-Enabled CMOS Photonics Platform. IEEE J. Select. Top. Quantum Electron..

[5] Kim I., Martins R.J., Jang J., Badloe T., Khadir S., Jung H.Y., Kim H., Kim J., Genevet P., Rho J. (2021). Nanophotonics for Light Detection and Ranging Technology. Nat. Nanotechnol..

[6] Poulton C.V., Yaacobi A., Cole D.B., Byrd M.J., Raval M., Vermeulen D., Watts M.R. (2017). Coherent Solid-State LIDAR with Silicon Photonic Optical Phased Arrays. Opt. Lett..

[7] Rogers C., Piggott A.Y., Thomson D.J., Wiser R.F., Opris I.E., Fortune S.A., Compston A.J., Gondarenko A., Meng F., Chen X. (2021). A Universal 3D Imaging Sensor on a Silicon Photonics Platform. Nature.

[8] Suyama S., Ito H., Kurahashi R., Abe H., Baba T. (2021). Doppler Velocimeter and Vibrometer FMCW LiDAR with Si Photonic Crystal Beam Scanner. Opt. Express.

[9] Poulton C.V., Byrd M.J., Russo P., Timurdogan E., Khandaker M., Vermeulen D., Watts M.R. (2019). Long-Range LiDAR and Free-Space Data Communication With High-Performance Optical Phased Arrays. IEEE J. Select. Top. Quantum Electron..

[10] Su T., Scott R.P., Djordjevic S.S., Fontaine N.K., Geisler D.J., Cai X., Yoo S.J.B. (2012). Demonstration of Free Space Coherent Optical Communication Using Integrated Silicon Photonic Orbital Angular Momentum Devices. Opt. Express.

[11] Rhee H.W., You J.B., Yoon H., Han K., Kim M., Lee B.G., Kim S.C., Park H.H. (2020). 32 Gbps Data Transmission With 2D Beam-Steering Using a Silicon Optical Phased Array. IEEE Photonics Technol. Lett..

[12] Miller S.A., Chang Y.C., Phare C.T., Shin M.C., Zadka M., Roberts S.P., Stern B., Ji X., Mohanty A., Jimenez Gordillo O.A. (2020). Large-Scale Optical Phased Array Using a Low-Power Multi-Pass Silicon Photonic Platform. Optica.

[13] Poulton C.V., Byrd M.J., Moss B., Timurdogan E., Millman R., Watts M.R. 8192-Element Optical Phased Array with 100° Steering Range and Flip-Chip CMOS. Proceedings of the Conference on Lasers and Electro-Optics, OSA.

[14] Chung S., Abediasl H., Hashemi H. (2018). A Monolithically Integrated Large-Scale Optical Phased Array in Silicon-on-Insulator CMOS. IEEE J. Solid-State Circuits.

[15] Hulme J.C., Doylend J.K., Heck M.J.R., Peters J.D., Davenport M.L., Bovington J.T., Coldren L.A., Bowers J.E. (2015). Fully Integrated Hybrid Silicon Two Dimensional Beam Scanner. Opt. Express.

[16] Acoleyen K.V., Bogaerts W., Jágerská J., Thomas N.L., Houdré R., Baets R. (2009). Off-Chip Beam Steering with a One-Dimensional Optical Phased Array on Silicon-on-Insulator. Opt. Lett. OL.

[17] Mohanty A., Li Q., Tadayon M.A., Roberts S.P., Bhatt G.R., Shim E., Ji X., Cardenas J., Miller S.A., Kepecs A. (2020). Reconfigurable Nanophotonic Silicon Probes for Sub-Millisecond Deep-Brain Optical Stimulation. Nat. Biomed. Eng..

[18] Mehta K.K., Bruzewicz C.D., McConnell R., Ram R.J., Sage J.M., Chiaverini J. (2016). Integrated Optical Addressing of an Ion Qubit. Nat. Nanotechnol..

[19] Giewont K., Hu S., Peng B., Rakowski M., Rauch S., Rosenberg J.C., Sahin A., Stobert I., Stricker A., Nummy K. (2019). 300-mm Monolithic Silicon Photonics Foundry Technology. IEEE J. Select. Top. Quantum Electron..

[20] Soref R.A., Bennett B. (1987). Electrooptical Effects in Silicon. IEEE J. Quantum Electron..

[21] Reed G.T., Mashanovich G., Gardes F.Y., Thomson D.J. (2010). Silicon Optical Modulators. Nat. Photonics.

[22] Cocorullo G., Rendina I. (1992). Thermo-Optical Modulation at 1.5 μm in Silicon Etalon. Electron. Lett..

[23] Watts M.R., Sun J., DeRose C., Trotter D.C., Young R.W., Nielson G.N. (2013). Adiabatic Thermo-Optic Mach–Zehnder Switch. Opt. Lett..

[24] Baets R., Bogaerts W., Kuyken B., Rahim A., Roelkens G., Spuesens T., Campenhout J.V., Thourhout D.V., Venghaus H., Grote N. (2017). Silicon Photonic Integrated Circuits. Fibre Optic Communication.

[25] Smalley D.E., Nygaard E., Squire K., Van Wagoner J., Rasmussen J., Gneiting S., Qaderi K., Goodsell J., Rogers W., Lindsey M. (2018). A Photophoretic-Trap Volumetric Display. Nature.

[26] Chun B.S., Kim K., Gweon D. (2009). Three-Dimensional Surface Profile Measurement Using a Beam Scanning Chromatic Confocal Microscope. Rev. Sci. Instrum..

[27] Fenn A.J., Temme D.H., Delaney W.P., Courtney W.E. (2000). The Development of Phased-Array Radar Technology. Linc. Lab. J..

[28] Sun J., Timurdogan E., Yaacobi A., Su Z., Hosseini E.S., Cole D.B., Watts M.R. (2014). Large-Scale Silicon Photonic Circuits for Optical Phased Arrays. IEEE J. Select. Top. Quantum Electron..

[29] Sun J., Timurdogan E., Yaacobi A., Hosseini E.S., Watts M.R. (2013). Large-Scale Nanophotonic Phased Array. Nature.

[30] Shin M.C., Mohanty A., Watson K., Bhatt G.R., Phare C.T., Miller S.A., Zadka M., Lee B.S., Ji X., Datta I. (2020). Chip-Scale Blue Light Phased Array. Opt. Lett..

[31] Zhang Y., Ling Y.C., Zhang K., Gentry C., Sadighi D., Whaley G., Colosimo J., Suni P., Yoo S.J.B. (2019). Sub-Wavelength-Pitch Silicon-Photonic Optical Phased Array for Large Field-of-Regard Coherent Optical Beam Steering. Opt. Express.

[32] Fukui T., Tanomura R., Komatsu K., Yamashita D., Takahashi S., Nakano Y., Tanemura T., Tanemura T. (2021). Non-Redundant Optical Phased Array. Optica.

[33] Hutchison D.N., Sun J., Doylend J.K., Kumar R., Heck J., Kim W., Phare C.T., Feshali A., Rong H. (2016). High-Resolution Aliasing-Free Optical Beam Steering. Optica.

[34] Kwong D., Hosseini A., Covey J., Zhang Y., Xu X., Subbaraman H., Chen R.T. (2014). On-Chip Silicon Optical Phased Array for Two-Dimensional Beam Steering. Opt. Lett..

[35] Fatemi R., Khachaturian A., Hajimiri A. (2019). A Nonuniform Sparse 2-D Large-FOV Optical Phased Array With a Low-Power PWM Drive. IEEE J. Solid-State Circuits.

[36] Chung S., Nakai M., Hashemi H. (2019). Low-Power Thermo-Optic Silicon Modulator for Large-Scale Photonic Integrated Systems. Opt. Express.

[37] Timurdogan E., Poulton C.V., Byrd M.J., Watts M.R. (2017). Electric Field-Induced Second-Order Nonlinear Optical Effects in Silicon Waveguides. Nat. Photonics.

[38] Zhang Z., Yu H., Huang Q., Zhou Z., Chen B., Dai T., Qiu H., Wang Y., Yang J. (2022). High-Speed and Low-Power Silicon Optical Phased Array Based on the Carrier-Depletion Mechanism. IEEE Photonics Technol. Lett..

[39] Xie W., Komljenovic T., Huang J., Tran M., Davenport M., Torres A., Pintus P., Bowers J. (2019). Heterogeneous Silicon Photonics Sensing for Autonomous Cars [Invited]. Opt. Express.

[40] Kang G., Youn C.H., Yu K., Park H.H., Kim S.H., You J.B., Lee D.S., Yoon H., Ha Y.G., Kim J.H. (2019). Silicon-Based Optical Phased Array Using Electro-Optic p-i-n Phase Shifters. IEEE Photonics Technol. Lett..

[41] Phare C.T., Shin M.C., Miller S.A., Stern B., Lipson M. (2018). Silicon Optical Phased Array with High-Efficiency Beam Formation over 180 Degree Field of View. arXiv.

[42] Jahani S., Kim S., Atkinson J., Wirth J.C., Kalhor F., Noman A.A., Newman W.D., Shekhar P., Han K., Van V. (2018). Controlling Evanescent Waves Using Silicon Photonic All-Dielectric Metamaterials for Dense Integration. Nat. Commun..

[43] Inoue D., Ichikawa T., Kawasaki A., Yamashita T. (2019). Demonstration of a New Optical Scanner Using Silicon Photonics Integrated Circuit. Opt. Express.

[44] Chang Y.C., Shin M.C., Phare C.T., Miller S.A., Shim E., Lipson M. (2021). 2D Beam Steerer Based on Metalens on Silicon Photonics. Opt. Express.

[45] López J.J., Skirlo S.A., Kharas D., Sloan J., Herd J., Juodawlkis P., Soljačić M., Sorace-Agaskar C. Planar-Lens Enabled Beam Steering for Chip-Scale LIDAR. Proceedings of the Conference on Lasers and Electro-Optics, OSA.

[46] Li C., Cao X., Wu K., Li X., Chen J. (2019). Lens-Based Integrated 2D Beam-Steering Device with Defocusing Approach and Broadband Pulse Operation for Lidar Application. Opt. Express.

[47] Kim S., Sloan J., López J.J., Kharas D., Herd J., Bramhavar S., Juodawlkis P., Barbastathis G., Johnson S., Sorace-Agaskar C. Luneburg Lens for Wide-Angle Chip-Scale Optical Beam Steering. Proceedings of the Conference on Lasers and Electro-Optics, OSA.

[48] Komljenovic T., Pintus P. (2018). On-Chip Calibration and Control of Optical Phased Arrays. Opt. Express.

[49] Li L.J., Chen W., Zhao X.Y., Sun M.J. (2019). Fast Optical Phased Array Calibration Technique for Random Phase Modulation LiDAR. IEEE Photonics J..

[50] Zhang H., Zhang Z., Peng C., Hu W. (2020). Phase Calibration of On-Chip Optical Phased Arrays via Interference Technique. IEEE Photonics J..

[51] Chrostowski L., Hochberg M. (2015). Silicon Photonics Design.

[52] Ito H., Kusunoki Y., Maeda J., Akiyama D., Kodama N., Abe H., Tetsuya R., Baba T. (2020). Wide Beam Steering by Slow-Light Waveguide Gratings and a Prism Lens. Optica.

[53] Miller S.A., Phare C.T., Chang Y.C., Ji X., Gordillo O.A.J., Mohanty A., Roberts S.P., Shin M.C., Stern B., Zadka M. 512-Element Actively Steered Silicon Phased Array for Low-Power LIDAR. Proceedings of the CLEO, Optical Society of America.

[54] Zadka M., Chang Y.C., Mohanty A., Phare C.T., Roberts S.P., Lipson M. (2018). On-Chip Platform for a Phased Array with Minimal Beam Divergence and Wide Field-of-View. Opt. Express.

[55] Dostart N., Zhang B., Khilo A., Brand M., Al Qubaisi K., Onural D., Feldkhun D., Wagner K.H., Popović M.A. (2020). Serpentine Optical Phased Arrays for Scalable Integrated Photonic Lidar Beam Steering. Optica.

[56] Ito H., Kusunoki Y., Akiyama D., Tetsuya R., Abe H., Baba T., Reed G.T., Knights A.P. (2019). Enhanced Light Emission from a Si Photonics Beam Steering Device Consisting of Asymmetric Photonic Crystal Waveguide. Proceedings of the Silicon Photonics XIV, San Francisco, CA, USA, 4–6 February 2019.

[57] Kondo K., Tatebe T., Hachuda S., Abe H., Koyama F., Baba T. (2017). Fan-Beam Steering Device Using a Photonic Crystal Slow-Light Waveguide with Surface Diffraction Grating. Opt. Lett..

[58] Ito H., Tatebe T., Abe H., Baba T. (2018). Wavelength-Division Multiplexing Si Photonic Crystal Beam Steering Device for High-Throughput Parallel Sensing. Opt. Express.

[59] Riemensberger J., Lukashchuk A., Karpov M., Weng W., Lucas E., Liu J., Kippenberg T.J. (2020). Massively Parallel Coherent Laser Ranging Using a Soliton Microcomb. Nature.

[60] Absil P.P., De Heyn P., Chen H., Verheyen P., Lepage G., Pantouvaki M., De Coster J., Khanna A., Drissi Y., Van Thourhout D., Reed G.T., Watts M.R. Imec ISiPP25G Silicon Photonics: A Robust CMOS-Based Photonics Technology Platform. Proceedings of the SPIE.

[61] Aflatouni F., Abiri B., Rekhi A., Hajimiri A. (2015). Nanophotonic Coherent Imager. Opt. Express.

[62] Fukui T., Kohno Y., Tang R., Nakano Y., Tanemura T. (2021). Single-Pixel Imaging Using Multimode Fiber and Silicon Photonic Phased Array. J. Lightwave Technol..

[63] Kohno Y., Komatsu K., Tang R., Ozeki Y., Nakano Y., Tanemura T. (2019). Ghost Imaging Using a Large-Scale Silicon Photonic Phased Array Chip. Opt. Express.

[64] Kuo P.C., Kuo S.I., Wang J.W., Jian Y.H., Ahmad Z., Fu P.H., Chang C., Shi J.W., Huang D.W., Liu Y. Actively Steerable Integrated Optical Phased Array (OPA) for Optical Wireless Communication (OWC). Proceedings of the 2022 Optical Fiber Communications Conference and Exhibition (OFC).

[65] Sun X., Zhang L., Zhang Q., Zhang W. (2019). Si Photonics for Practical LiDAR Solutions. Appl. Sci..

[66] Heck M.J.R. (2017). Highly Integrated Optical Phased Arrays: Photonic Integrated Circuits for Optical Beam Shaping and Beam Steering. Nanophotonics.

[67] Raj T., Hashim F.H., Huddin A.B., Ibrahim M.F., Hussain A. (2020). A Survey on LiDAR Scanning Mechanisms. Electronics.

[68] Royo S., Ballesta-Garcia M. (2019). An Overview of Lidar Imaging Systems for Autonomous Vehicles. Appl. Sci..

[69] Haus H.A. (1984). Waves and Fields in Optoelectronics.

[70] Kim S., Westly D.A., Roxworthy B.J., Li Q., Yulaev A., Srinivasan K., Aksyuk V.A. (2018). Photonic Waveguide to Free-Space Gaussian Beam Extreme Mode Converter. Light Sci. Appl..

[71] Ginel-Moreno P., Sánchez-Postigo A., de-Oliva-Rubio J., Hadij-ElHouati A., Ye W.N., Wangüemert-Pérez J.G., Molina-Fernández Í., Schmid J.H., Cheben P., Ortega-Moñux A. (2021). Millimeter-Long Metamaterial Surface-Emitting Antenna in the Silicon Photonics Platform. Opt. Lett..

[72] Raval M., Poulton C.V., Watts M.R. (2017). Unidirectional Waveguide Grating Antennas with Uniform Emission for Optical Phased Arrays. Opt. Lett..

[73] Yulaev A., Zhu W., Zhang C., Westly D.A., Lezec H.J., Agrawal A., Aksyuk V. (2019). Metasurface-Integrated Photonic Platform for Versatile Free-Space Beam Projection with Polarization Control. ACS Photonics.

[74] Hsieh P.Y., Zhao Y., Hsu C.Y., Shin M.C., Phare C.T., Miller S.A., Shim E., Lipson M., Chang Y.C. Metasurface on Silicon Photonics for Beam Steering and Focusing. Proceedings of the 26th Optoelectronics and Communications Conference, OSA.

[75] Ding Y., Chen X., Duan Y., Huang H., Zhang L., Chang S., Guo X., Ni X. (2022). Metasurface-Dressed Two-Dimensional on-Chip Waveguide for Free-Space Light Field Manipulation. ACS Photonics.

[76] Notaros J., Poulton C.V., Raval M., Watts M.R. (2018). Near-Field-Focusing Integrated Optical Phased Arrays. J. Lightwave Technol..

[77] Guo X., Ding Y., Chen X., Duan Y., Ni X. (2020). Molding Free-Space Light with Guided Wave–Driven Metasurfaces. Sci. Adv..

[78] Yu S., Lu J., Ginis V., Kheifets S., Lim S.W.D., Qiu M., Gu T., Hu J., Capasso F. (2021). On-Chip Optical Tweezers Based on Freeform Optics. Optica.

[79] Mehta K.K., Ram R.J. (2017). Precise and Diffraction-Limited Waveguide-to-Free-Space Focusing Gratings. Sci Rep..

[80] Zhao Z., Fan S. (2020). Design Principles of Apodized Grating Couplers. J. Lightwave Technol..

[81] Planchon T.A., Gao L., Milkie D.E., Davidson M.W., Galbraith J.A., Galbraith C.G., Betzig E. (2011). Rapid Three-Dimensional Isotropic Imaging of Living Cells Using Bessel Beam Plane Illumination. Nat. Methods.

[82] Notaros J., Poulton C.V., Byrd M.J., Raval M., Watts M.R. (2017). Integrated Optical Phased Arrays for Quasi-Bessel-Beam Generation. Opt. Lett..

[83] Rubinsztein-Dunlop H., Forbes A., Berry M.V., Dennis M.R., Andrews D.L., Mansuripur M., Denz C., Alpmann C., Banzer P., Bauer T. (2017). Roadmap on Structured Light. J. Opt..

[84] Cai X., Wang J., Strain M.J., Johnson-Morris B., Zhu J., Sorel M., O’Brien J.L., Thompson M.G., Yu S. (2012). Integrated Compact Optical Vortex Beam Emitters. Science.

[85] Liu J., Li S.M., Zhu L., Wang A.D., Chen S., Klitis C., Du C., Mo Q., Sorel M., Yu S.Y. (2018). Direct Fiber Vector Eigenmode Multiplexing Transmission Seeded by Integrated Optical Vortex Emitters. Light Sci. Appl..

[86] Schulz S.A., Machula T., Karimi E., Boyd R.W. (2013). Integrated Multi Vector Vortex Beam Generator. Opt. Express.

[87] Zhu J., Cai X., Chen Y., Yu S. (2013). Theoretical Model for Angular Grating-Based Integrated Optical Vortex Beam Emitters. Opt. Lett..

[88] Shao Z., Zhu J., Zhang Y., Chen Y., Yu S. (2018). On-Chip Switchable Radially and Azimuthally Polarized Vortex Beam Generation. Opt. Lett..

[89] Lin Y., Mak J.C.C., Chen H., Mu X., Stalmashonak A., Jung Y., Luo X., Lo P.G.-Q., Sacher W.D., Poon J.K.S. (2021). Low-Loss Broadband Bi-Layer Edge Couplers for Visible Light. Opt. Express.

[90] Yong Z., Chen H., Luo X., Govdeli A., Chua H., Azadeh S.S., Stalmashonak A., Lo G.-Q., Poon J.K.S., Sacher W.D. (2022). Power-Efficient Silicon Nitride Thermo-Optic Phase Shifters for Visible Light. Opt. Express.

[91] Turk N., Raza A., Wuytens P., Demol H., Van Daele M., Detavernier C., Skirtach A., Gevaert K., Baets R. (2019). Comparison of Free-Space and Waveguide-Based SERS Platforms. Nanomaterials.

[92] Shim E., Chen Y., Masmanidis S., Li M. (2016). Multisite Silicon Neural Probes with Integrated Silicon Nitride Waveguides and Gratings for Optogenetic Applications. Sci. Rep..

[93] Sacher W.D., Chen F.D., Moradi-Chameh H., Luo X., Fomenko A., Shah P., Lordello T., Liu X., Almog I.F., Straguzzi J.N. (2021). Implantable Photonic Neural Probes for Light-Sheet Fluorescence Brain Imaging. Neurophotonics.

[94] Lin N.C., Zhao X., Hassan S., Raghuram A., Veeraraghavan A., Robinson J.T. Implantable Integrated Photonic Probe for Light Sheet Brain Imaging in Vivo. Proceedings of the Biophotonics Congress 2021, OSA.

[95] Mehta K.K., Zhang C., Malinowski M., Nguyen T.L., Stadler M., Home J.P. (2020). Integrated Optical Multi-Ion Quantum Logic. Nature.

[96] Ji X., Roberts S., Corato-Zanarella M., Lipson M. (2021). Methods to Achieve Ultra-High Quality Factor Silicon Nitride Resonators. APL Photonics.

[97] Vermeulen D., Selvaraja S., Verheyen P., Lepage G., Bogaerts W., Absil P., Van Thourhout D., Roelkens G. (2010). High-Efficiency Fiber-to-Chip Grating Couplers Realized Using an Advanced CMOS-Compatible Silicon-On-Insulator Platform. Opt. Express.

[98] Cardenas J., Poitras C.B., Luke K., Luo L.W., Morton P.A., Lipson M. (2014). High Coupling Efficiency Etched Facet Tapers in Silicon Waveguides. IEEE Photonics Technol. Lett..

